# Supplementary material for: E-Cigarette Aerosols Promote Oral S. aureus Colonization by Delaying an Immune Response and Bacterial Clearing
Source: Cells. 2022 Feb 23;11(5):773. doi: 10.3390/cells11050773 (PMC8909134; doi:10.3390/cells11050773)
Supplement: Supplementary file 1 [file cells-11-00773-s001.zip › cells-1586961-supplementary.pdf]

Supplementary Materials: The following are available online at www.mdpi.com/xxx/s1,

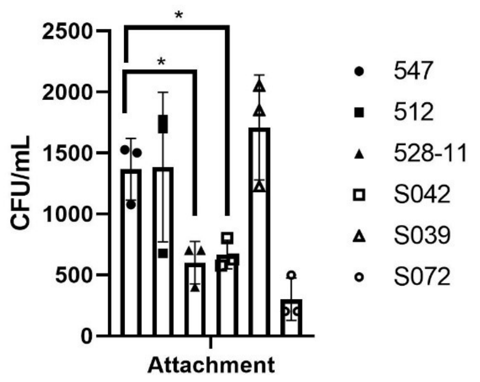

Figure S1. The attachment capacity varies between *S. aureus s*trains and sequence types. *S. aureus* strains from smokers (S042, S039, S072) and non-smokers (547,512,528-11) were co-cultured with OKF6 cells for 3 hours (MOI 10^{-3}), attached bacteria was serially diluted and plated.

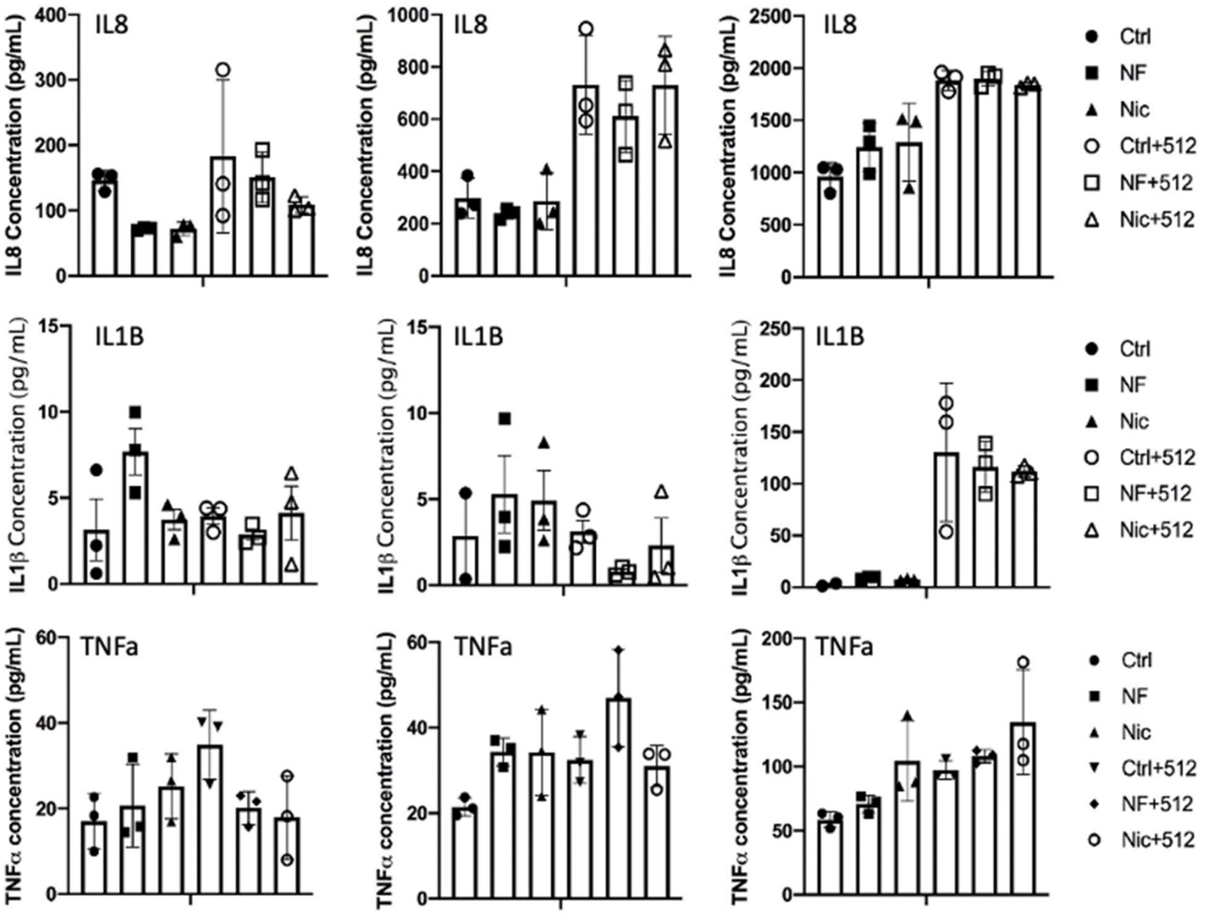

Figure S2. S. aureus does induce an epithelial inflammatory response when co-cultured (MOI 10-3) with epithelial oral cells OKF6. The release of IL8, IL1β and TNFα were measured by ELISA of conditioned media from OKF6 cells pre-exposed to E-cig aerosol 30 min (6 puffs 30 second interval), allowed to recover for 24 hours, followed by co-cultured with S. aureus 512

at MOI 10-3). Conditioned media was collected and analyzed at 3 hours (left panels), 7 hours (middle panels), and 24 hours (right panels).

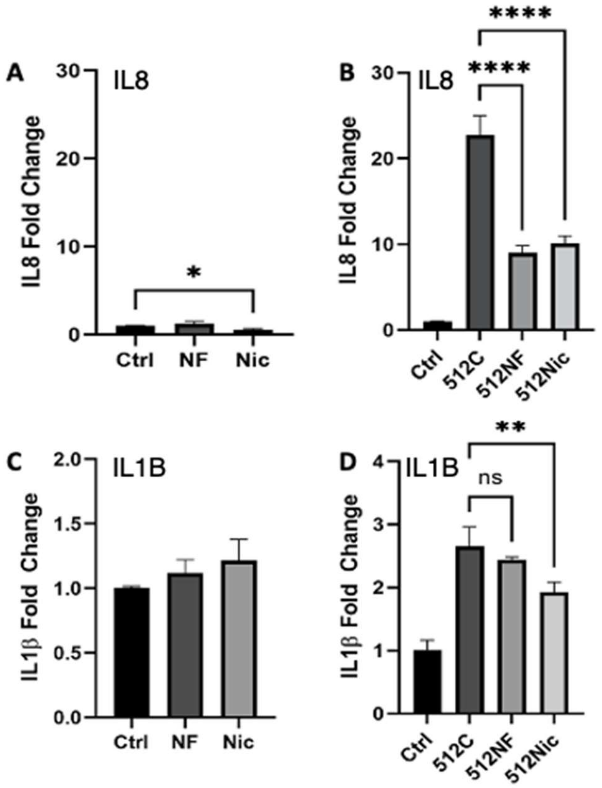

Figure S3. E-cig aerosol decreased the expression of proinflammatory cytokines IL8 and IL1β. OKF6 cells were pre-exposed to e-cig aerosol and co-cultured with S. aureus 512 (MOI 10-2). Expression of the proinflammatory cytokines IL8 (A,B), and IL1β (C,D) was measured by qRT-PCR. * P<0.0001.
